# Health-relevant personality traits are associated with measures of health, well-being, stress and psychosocial work environment over time

**DOI:** 10.1371/journal.pone.0314321

**Published:** 2024-12-13

**Authors:** Karin Villaume, Susanne Tafvelin, Dan Hasson

**Affiliations:** 1 Department Learning Informatics Management and Ethics, Medical Management Centre, Karolinska Institutet, Stockholm, Sweden; 2 Stress Clinic (Stiftelsen Stressmottagningen), Stockholm, Sweden; 3 Mayo Clinic, Scottsdale, Arizona, United States of America; 4 Department of Psychology, Umeå University, Umeå, Sweden; University of Glasgow, UNITED KINGDOM OF GREAT BRITAIN AND NORTHERN IRELAND

## Abstract

Trends in health, well-being, stress and the psychosocial work environment were studied using repeated measurements over time. The role of health-relevant personality in predicting development over time and typical ratings was also assessed. 517 individuals were included. Health-relevant personality 5 inventory (HP5i) was used to assess personality: HW-11 was used for repeated assessment of outcome measures. There were clear seasonal variations over time. Multilevel growth curves demonstrated that some changes over time could partly be explained by higher levels of negative affectivity at baseline. Individuals’ typical ratings were predicted by health-relevant personality. Those with higher negative affectivity typically perceived worse health, well-being and psychosocial work environment. Health-relevant personality seems to be associated with changes in health, well-being, stress and the psychosocial work environment over time. The findings highlight the importance of regular assessments of these indicators as they clearly vary over time and the trends seem to follow seasonal patterns.

## Introduction

A popular approach in organizational development and occupational health promotion is to utilize questionnaires to assess perceptions of health and the psychosocial work environment [1]. Based on the outcomes, action plans and interventions can be designed and implemented as a way of improving the work environment systematically. Hence, the starting point for continuous organizational improvements is often based on current occupational and psychosocial circumstances.

One of many challenges is that these measures are typically assessed retrospectively, meaning that the employees rate how the variables of interest have been during the past month or year, for instance. Retrospective ratings have some shortcomings, primarily the risk for recall bias, which is a systematic error in recalling the past accurately [2]. Combined with other confounders and forms of bias, as well as validity issues for questionnaires, results can potentially be misleading [1]. The current situation when filling out the questionnaire most likely influences the responses [3, 4]. Indeed, the current state has been found to be strongly associated with retrospective survey responses [5]. In that study, ratings of health, stress, concentration ability, energy level and sleep quality last night correlated strongly with the ratings of the same variables for last month (last 6 months for sleep) and last year. Furthermore, variables such as perceived stress and mood typically change over time [6], which implies the importance of regular assessments for valid interpretations. This conclusion is further supported by studies that demonstrate seasonal variations in questionnaire responses over time [7, 8]. These variations seem to follow a seasonal flow in cyclic and non-random patterns. In a study by Smith [8], happiness was reported to be highest during the spring, to slowly decline during the summer and fall, reaching a low in the winter and increasing during the following spring. Similarly, seasonal variations are also found in the secretion of some hormones [9, 10] and blood pressure [11, 12]. The stress hormone cortisol for instance, tends to increase during the fall, indicating higher stress levels, and decrease again during the spring, indicating lower levels of stress. Thus, the current state and seasonal variations may confound results of occupational work environment and health surveys. This clearly raises questions as to how accurate assessments are when only measured occasionally or once a year and when variables are rated retrospectively.

Another possible confounder might be individual characteristics, such as personality. By now, it is well established that personality is associated with health [13, 14] and perceptions of the psychosocial work environment [15], which will be described below. However, it is still not known if and how personality is associated with such variables when assessed over time, or if personality can predict change over time. The present study aims to investigate this further.

### Personality in relation to health and ill-health

The relationships between personality and health are complex and multidimensional [13, 14]. The most consistent findings are that higher levels of neuroticism or negative affectivity are associated with reports of poorer health and more physical and mental symptoms compared to those with lower levels of those traits [13, 14, 16–29]. The well-known Terman Life Cycle Study, where individuals were followed from age 11 until their deaths, has yielded impressive insights about the relationship between personality and health over a life course [30]. Neuroticism was found to be the strongest predictor of poor perceived health [19], but the mechanisms behind these outcomes have not yet fully been established. Several models have been presented that may partly explain the associations between personality and health. The most common model involves stress-moderation mechanisms [31]. Briefly described, this model implies that the repeated stress responses and coping strategies people use can increase or decrease the vulnerability for ill-health and disease. A recent longitudinal study found that people with higher levels of neuroticism typically tend to react with more negative emotion to daily stressors, compared to people with lower levels of neuroticism [26]. This is thus in line with the model involving stress-moderation mechanisms, but other models have also been proposed. The causal links between neuroticism, health and ill-health have thus not been completely established and this is most likely due to the multi-dimensional nature of the associations. The results can be confounded by environmental, situational, hereditary and social factors, to mention some, making it difficult to infer causality.

A stronger and more consistent causal link has however been found for individuals who are socially stable and highly conscientious [30]. These individuals are less likely to exhibit poor health, both physically and mentally, compared to individuals with lower levels of that trait [21]. The more direct causality could be explained by the fact that highly conscientious individuals tend to lead more stable lives. They have more predictable routines, stable social support and tend to engage in healthy behaviors. Even though there are numerous publications about personality and health, general conclusions are difficult to draw. There are several reasons for this, not least because health problems and personality are assessed in different ways in various studies. Other explanations include varying characteristics of the samples, e.g., ranging from large populations to small samples, healthy individuals, patients, elderly and young students, etc. Moreover, some studies ask the individual to think back as much as 12 months to remember symptoms or disease prevalence. As mentioned above, such retrospective approaches are most likely influenced by recall bias [6, 32].

### Personality and perceived psychosocial work environment

Alongside the health and personality research, the interest to better understand personality in relation to the psychosocial work environment has flourished [15]. For instance, studies have found personality to be associated with perceived job stressors and strains [33–35]. More specifically, higher level of negative affectivity is associated with higher levels of perceived job stress. Some studies have found personality to be associated with perceived job satisfaction [36–40]. Higher levels of neuroticism and negative affectivity predict lower job satisfaction and higher levels of conscientiousness and extraversion predict higher job satisfaction. These studies have yielded valuable information about personality and aspects of the psychosocial work environment but leave some important questions unanswered. There is clearly a need to better understand how indicators of stress, health and the psychosocial work environment are perceived by persons with different personality traits when measured repeatedly over time. It is also interesting to know if these findings are consistent and create predictable patterns over time. Modern technology, like web-based questionnaires, offer opportunities for regular and frequent assessments. To our knowledge, no studies have investigated personality in relation to perceived health and the psychosocial work environment during an occupational health intervention, using intensive, repeated monitoring over time. It is plausible that such monitoring over time will yield a more reliable foundation for continuous organizational improvements, compared to measurements at isolated time points.

### Aim

The aim of this study is to investigate personality traits in relation to health-related variables and indicators of the psychosocial work environment using repeated, intensive assessments over time. Moreover, the aim is to investigate if personality traits predict changes in perceptions of health and psychosocial work environment over time.

## Materials and methods

This study uses data from an occupational health and stress management intervention that targeted staff employed at primary schools in Stockholm, Sweden. The aim of the intervention was to promote health and the psychosocial work environment by providing access to a web-based tool. The tool consisted of 11 global screening questions (described in detail below) that could be used as frequently as the participants wanted, although recommended at least once a week. The 11-item brief survey took approximately 15–30 seconds to answer. The respondents received instant, automated feedback and were able to monitor their development over time. The tool also included 18 self-help exercises within areas such as stress management, relaxation and recovery, goal setting, emotional control and body awareness. There was a web-based diary for expressive writing as well as scientific news articles regarding health. A previous version of the tool has been evaluated before, in a randomized controlled trial. Beneficial and systematic effects were found in both biomarkers and questionnaires [41]. During the intervention, four more extensive web-based surveys were distributed. These surveys consisted of approximately 250–300 questions within areas such as health, lifestyle, personality (described below), psychosocial work environment and leadership. The study participants received instant, automated feedback on their results and were offered to be contacted by the occupational healthcare provider if they wanted to.

### Participants

The participants were recruited after a thorough implementation process established with the Human Resources department, labor union representatives and management. A total of 21 schools enrolled in the study and all employees were invited to participate in the intervention. Out of 2,090 individuals, 1,001 (48%) signed up for the web-based tool sometime during the study period (May 2014 –December 2015). For the purpose of the present study, we selected only the participants who had responded to the baseline health-relevant personality items (described below) as well to the 11-item health and psychosocial work environment items at least once. This yielded a sample of 517 participants that were included in the analyses. The web-based intervention was available to the participants during 20 months between 2014 and 2015. Participation in the intervention was voluntary and all study participants provided their written informed consent. The ethical committee in Stockholm, Sweden approved the study in full (Protocol approval number: 2014/274-31/5).

### Measures

The following measures were used in the analyses.

#### Personality

Health-relevant personality traits were assessed in the beginning of the intervention using the Health-Relevant Personality 5 inventory (HP5i) [22]. It consists of 20 items aiming to capture facets of the Five Factor Model (FFM). HP5i was chosen for the following reasons;

1. The HP5i was initially developed to be used in large-scale health studies and has shown acceptable psychometric properties. It was therefore considered to be suitable for the present study. The shortness of the inventory (20 items) was also desirable, since the participants in the present study had very limited time to respond to extensive surveys.
2. As the project involved an occupational health promotion and stress management intervention, it seemed appropriate to assess health-relevant personality traits as opposed to more broad traits. Assessing specific personality traits have been found to be reliable measures that add unique information not accounted for when using broader dimensions [28, 42]. Thus, narrower traits have been recommended in studies assessing specific aspects of health, which the present study does.

The HP5i consists of the following five facets: Hedonic capacity (a facet of Extraversion), aims to assess the extent to which an individual is prone to engage in goal-directed behavior, enthusiasm and experience pleasure and enjoyment in life. Negative affectivity (a facet of Neuroticism) aims to assess the extent to which an in individual is prone to nervous tension and distress. Impulsivity (a facet of Conscientiousness) aims to capture the extent to which an individual takes the day as it comes without making plans, is unthoughtful and somewhat unreliable. Antagonism (a facet of Agreeableness) intends to capture the degree to which an individual is oppositional, argumentative and sarcastic. Lastly, Alexithymia (a facet of Openness) aims to assess the extent to which an individual is uninterested in talking about and understanding emotions and feelings [22, 43]. Responses were given on a 4-point Likert scale with the response alternatives “Does not apply at all”, “Does not apply very much”, “Applies pretty much”, “Applies completely”. The following Cronbach’s alpha coefficients were obtained for each index: Antagonism: .60, Impulsivity: .82, Hedonic capacity: .67, Negative affectivity: .60 and Alexithymia: .69.

#### Repeated measures of health and psychosocial work environment over time

A brief questionnaire, consisting of 11 global single items and called HW-11, was used to repeatedly assess perceptions of health, stress, well-being and the psychosocial work environment. The health-related items were; self-rated health (SRH), sleep quality, concentration ability, stress, energy level, control and social support. The psychosocial work environment items included; work efficiency, job satisfaction, workload and work atmosphere. All items were similar in phrasing with regards to assessing the current state. For instance, SRH was assessed by “How do you feel right now?”. Energy level was assessed by “How is your energy level right now?” Sleep quality was assessed by “How did you sleep last night?” etc. For nine items, responses were given on a verbal rating scales (VRS) with five descriptors. For two of the items (stress and work efficiency), responses were given on a visual analogue scale (VAS) with two anchors.

### Data analytic approach

The personality variables and the 11 outcome variables were tested for normality using the Kolmogorov Smirnov test. None of the variables passed this rather strict test. Spearman correlations were therefore performed to investigate possible associations between health-relevant personality and HW-11. Thereafter, data were plotted graphically in order to visualize possible trends in perceived health, stress and psychosocial work environment over time. For pedagogic reasons, the personality items were trichotomized based on quartiles or near quartiles to display low, medium or high levels of each personality trait in the plots. The median monthly rating of each indicator was used to visualize development over time.

In order to evaluate if health-relevant personality traits could predict the indicators of perceived health and psychosocial work environment over time, multilevel growth curves were performed. This approach was selected as it allows for the investigation of response patterns over time, where the number of responses differ between individuals. Data were treated as having a 2-level hierarchical structure. In Level-1 (within-person) changes over time in the outcome were modeled within each individual [44]. The underlying equation for this involves two individual growth parameters; the individual’s baseline, and the effect of time on the outcome. The effect of time represents an individual’s change over time, also known as a growth curve or trajectory. Intra-class correlations were calculated in order to assess the percentage of within-person variance. In Level-2 (between-person), the model examines how these individual changes over time may vary between individuals [45]. To assess cross-level interactions, meaning if personality traits could predict the growth parameters (e.g. development in health, sleep quality etc.), we included health-relevant personality traits as key predictors. Analyses were made with the software IBM SPSS version 25 and Mplus version 7.31. The method Full Information Maximum Likelihood (FIML), which is the default method in Mplus, was employed to account for missing values in the data. The level of statistical significance was set to .05.

## Results

**Fig 1:**
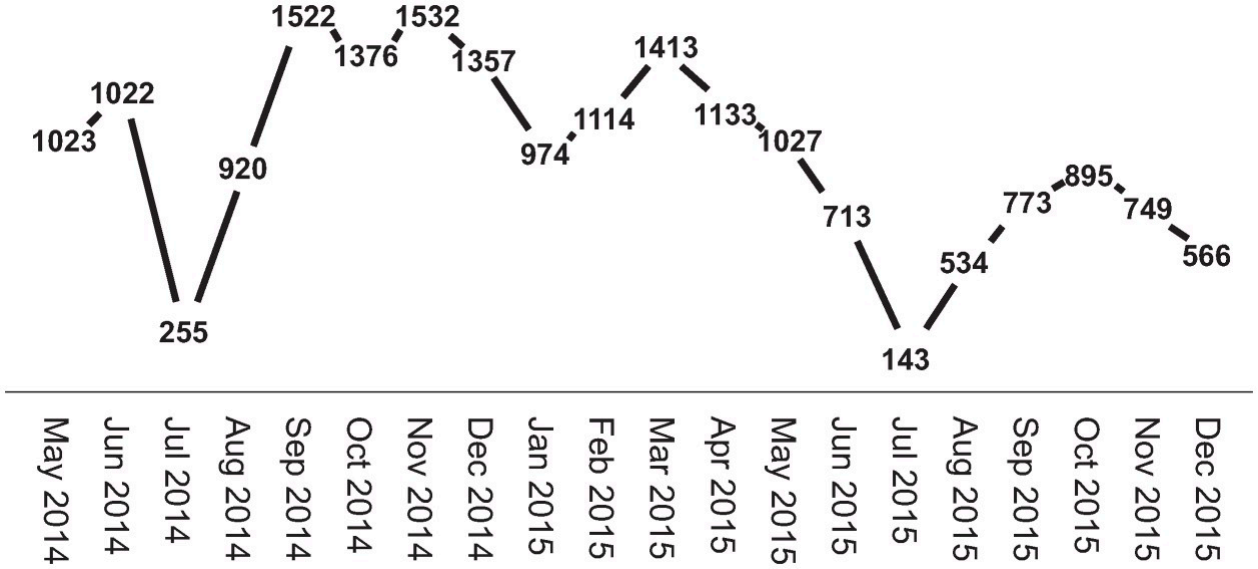
The total number of measurements per month, n = 517. Note that the number of responses given differ between participants.

The majority of the participants were women (81%; n = 418, 19% men; n = 99). Most (83%) had an academic degree and were married or in a relationship (78%). Mean age was 48 (SD ± 10 years), range 21–69 years. During the intervention, the average number of measurements were 25, median 15; range 1–241 measurements per individual. The number of measurements varied over time and ranged between 143 and 1,532 entries per month (Fig 1).

The descriptive statistics of the repeated HW-11 measures as well as correlation coefficients are presented in Table 1. Weak to moderate correlations were found between most health-relevant personality traits and the HW-11 items (r range -.017, p < 0.05–.291, p < 0.01). The exception, alexithymia, was negatively associated with only four out of the HW-11 variables, i.e., sleep, stress, workload and work atmosphere. Correlations between the HW-11 items ranged from weak to strong (r range; .023, p < 0.05 –.75, p < 0.01). The strongest correlations were found between energy and concentration ability (r = .75, p < 0.01), self-rated health and concentration ability (r = .74, p < 0.01) and energy and self-rated health (r = .70, p < 0.01).

**Table 1:** Mean values, standard deviations (SD), median, intra-class correlations (ICC) and Spearman correlations.

	**Mean**	**SD**	**Median**	**I-ICC**	**1. SRH**	**2. Sleep quality**	**3. Concentration ability**	**4. Stress**	**5. Energy level**	**6. Control**	**7. Social support**	**8. Work efficiency**	**9. Job satisfaction**	**10. Workload**	**11. Work atmosphere**	**A). Hedonic capacity**	**B). Negative affectivity**	**C). Antagonism**	**D). Impulsivity**
**1. SRH**	69.10	17.64	72	44.3%															
**2. Sleep quality**	67.10	21.30	71	37.3%	.576**														
**3. Concentration ability**	70.02	16.82	72	52.0%	.737**	.609**													
**4. Stress**	47.63	25.68	49	42.5%	-.290**	-.176**	-.253**												
**5. Energy level**	62.52	62.52	66	45.7%	.702**	.529**	.747**	-.118**											
**6. Control**	68.86	16.72	71	56.1%	.642**	.479**	.619**	-.286**	.573**										
**7. Social support**	70.68	18.28	73	67.0%	.521**	.395**	.477**	-.200**	.441**	.619**									
**8. Work efficiency**	67.58	19.95	71	41.5%	.480**	.375**	.614**	0.01	.599**	.460**	.403**								
**9. Job satisfaction**	64.53	19.64	68	55.6%	.660**	.469**	.664**	-.207**	.670**	.580**	.460**	.610**							
**10. Workload**	64.75	20.00	67	33.6%	-.034**	.011	.023**	.577**	.096**	-.045**	.034**	.298**	.080**						
**11. Work atmosphere**	68.77	17.24	71	55.5%	.492**	.341**	.479**	-.227**	.454**	.455**	.383**	.399**	.635**	.364					
**A). Hedonic capacity**	3.20	.40	3.25		.225**	.202**	.230**	-.060**	.189**	.235**	.291**	.208**	.235**	.098**	.194**				
**B). Negative affectivity**	2.22	.55	2.25		-.205**	-.188**	-.194**	.132**	-.177**	-.168**	-.206**	-.149**	-.174**	.005	-.137**	-.321**			
**C). Antagonism**	1.81	.49	1.75		-.086**	-.142**	-.072**	-.037**	-.066**	-.095**	-.099**	-.060**	-.077**	-.058**	-.064**	-.173**	.233**		
**D). Impulsivity**	2.02	.62	2.00		-.079**	-.108**	-.068**	-.028**	-.060**	-.090**	-.072**	-.068**	-.043**	-.073**	-.009	-.043**	.241**	.425**	
**E). Alexithymia**	1.79	.51	1.75		.001	-.049**	-.014	-.022**	.013	.010	.015	.007	-.015	-.017*	-.052**	-.197**	.018*	.383**	.109**

The ICC were calculated separately for each HW-11 variable to assess the percentage of within-person variance. Respondents, n = 517, number of assessments: 15,251–19,207.

* p < 0.05,

** p < 0.01.

The intra-class correlations (ICC) demonstrate the proportion of total variance that is attributed to the cluster level. In this case, the ICC represents the proportion of the variance that is explained by how an individual typically responds to each question. The explained variance for each HW-11 item ranged between 34% (for workload) and 67% (for social support). This indicates that the typical responses to the question about social support were more stable than the typical responses to the item about workload, where repeated responses seem to vary more on the individual level.

### Visualization of assessments over time

**Fig 2:**
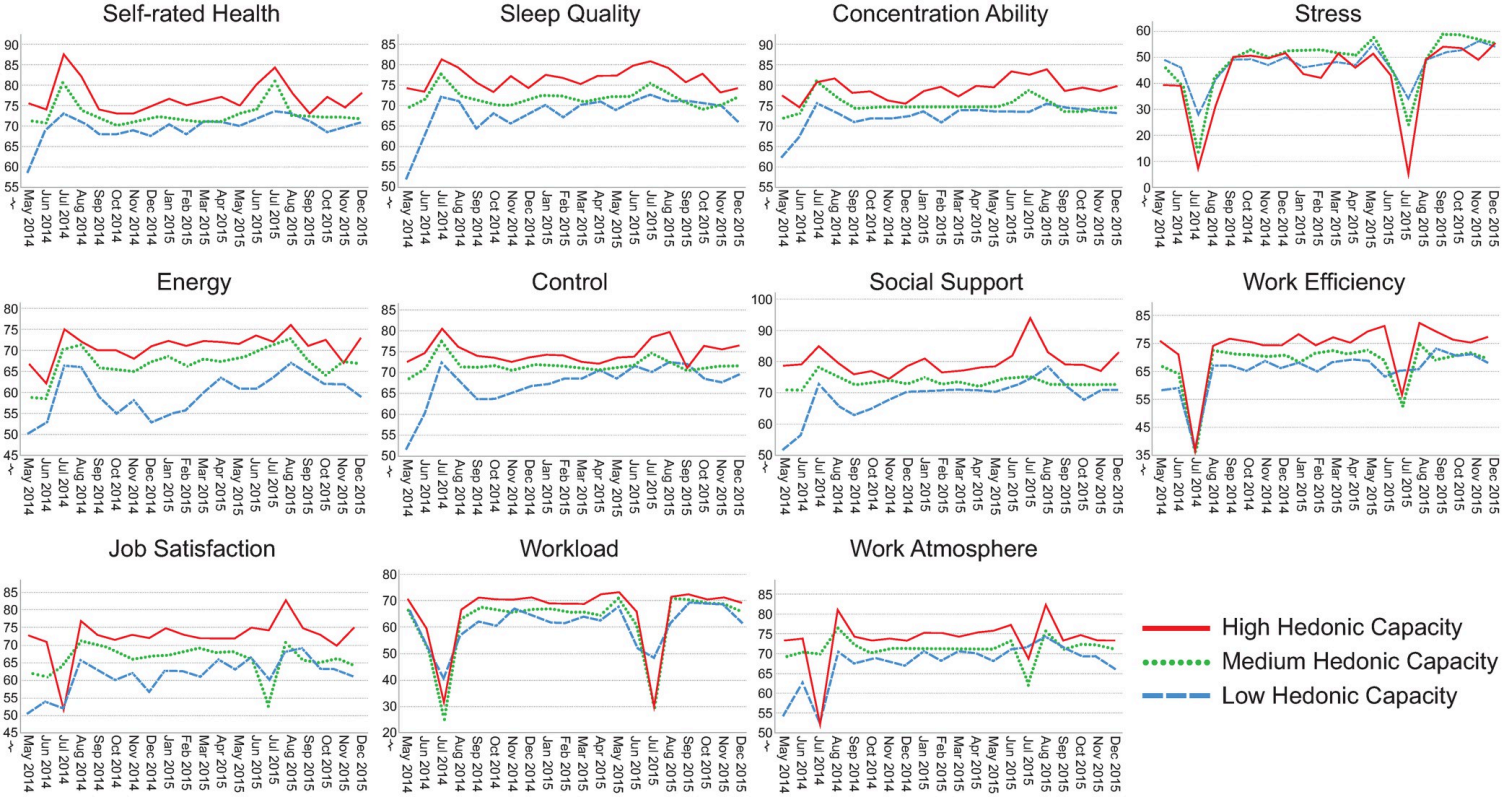
Median self-rated health, sleep quality, concentration ability, stress, energy, control, social support, work efficiency, job satisfaction, workload and work atmosphere plotted monthly from May 2014 –December 2015. The lines represent levels of hedonic capacity (low, median and high). Please note that the y-axes are broken for space saving purposes. N = 71–484 per month, number of assessments = 143–1,532 per month.

**Fig 3:**
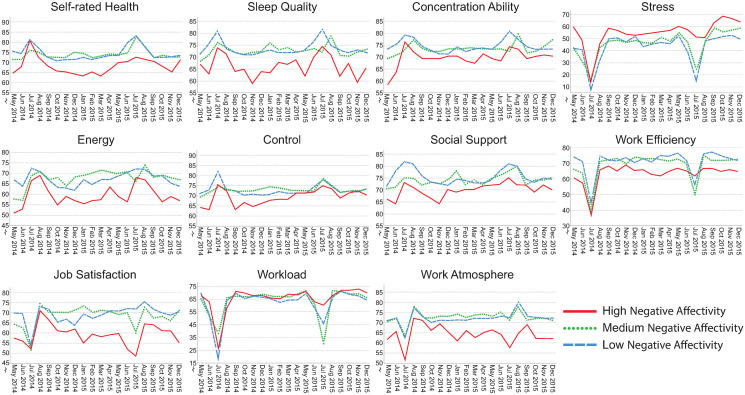
Median self-rated health, sleep quality, concentration ability, stress, energy, control, social support, work efficiency, job satisfaction, workload and work atmosphere plotted monthly from May 2014 –December 2015. The lines represent levels of negative affectivity (low, median and high). Please note that the y-axes are broken for space saving purposes. N = 71–484 per month, number of assessments = 143–1,532 per month.

**Fig 4:**
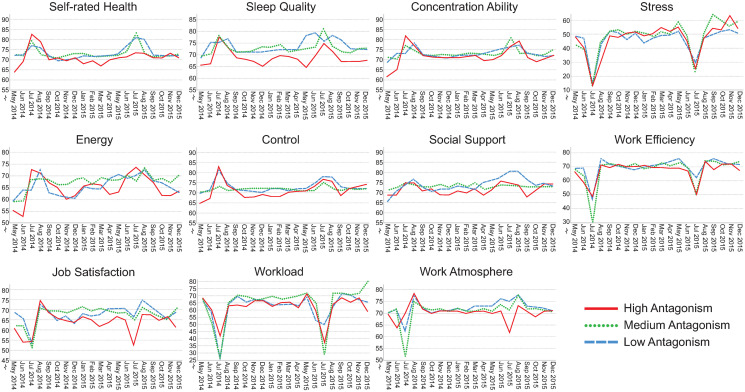
Median self-rated health, sleep quality, concentration ability, stress, energy, control, social support, work efficiency, job satisfaction, workload and work atmosphere plotted monthly from May 2014 –December 2015. The lines represent levels of antagonism (low, median and high). Please note that the y-axes are broken for space saving purposes. N = 71–484 per month, number of assessments = 143–1,532 per month.

**Fig 5:**
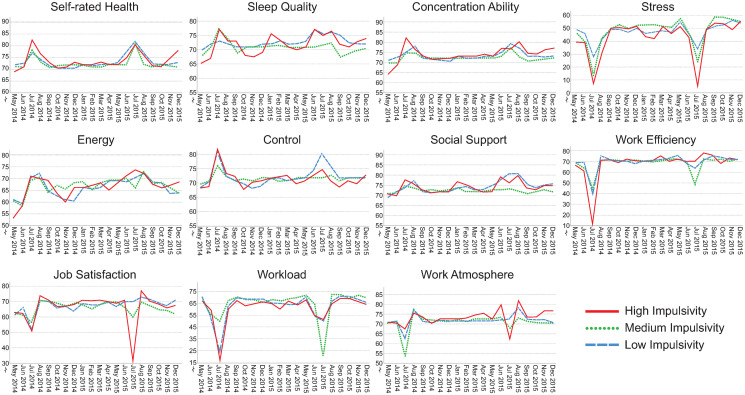
Median self-rated health, sleep quality, concentration ability, stress, energy, control, social support, work efficiency, job satisfaction, workload and work atmosphere plotted monthly from May 2014 –December 2015. The lines represent levels of impulsivity (low, median and high). Please note that the y-axes are broken for space saving purposes. N = 71–484 per month, number of assessments = 143–1,532 per month.

**Fig 6:**
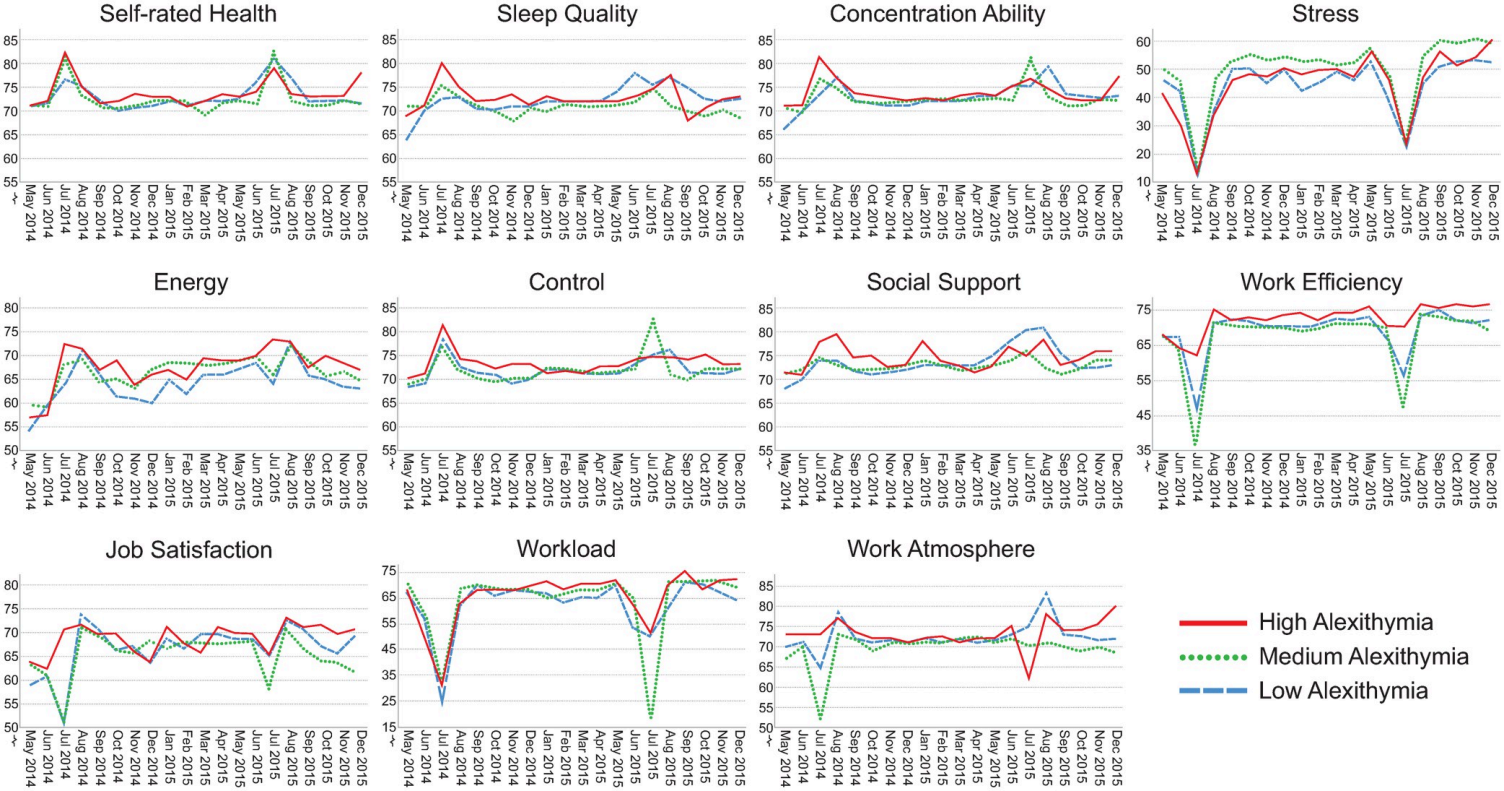
Median self-rated health, sleep quality, concentration ability, stress, energy, control, social support, work efficiency, job satisfaction, workload and work atmosphere plotted monthly from May 2014 –December 2015. The lines represent levels of alexithymia (low, median and high). Please note that the y-axes are broken for space saving purposes. N = 71–484 per month, number of assessments = 143–1,532 per month.

Figs 2–6 illustrate how the indicators of health, stress and well-being and of the psychosocial work environment develop monthly over the entire intervention period for each health-relevant personality trait. The graphs clearly demonstrate fluctuations in the indicators over time. Most health-related indicators improve during the summer months of June and July (when the majority of the participants were on vacation). The indicators worsen during the fall months of September, October and November before they start to improve again. For stress, work efficiency and workload, a distinct drop is seen in July, followed by peaks in August when most study participants came back to work after their vacation.

Although similar trends are present between those with low, medium and high levels of health-relevant personality traits, there seem to be differences in absolute rating levels. The most pronounced distinctions in absolute rating levels can be seen between those having low, medium and high levels of hedonic capacity and negative affectivity. Individuals with high levels of hedonic capacity seem to continuously rate better health, sleep quality, concentration ability, energy level, sense of control and higher satisfaction with social support compared to those having medium or low levels of that trait. They also seem to rate the psychosocial work environment indicators more positively and tend to perceive slightly less stress compared to those with medium or low levels. The reverse pattern is found for individuals with high levels of negative affectivity. They seem to continuously rate health-related aspects as worse, perceive more stress combined with a higher workload compared to those with medium or low levels of that trait. The differences in perceptions between low, medium and high levels of antagonism, impulsivity and alexithymia seem to be less pronounced.

### Health-relevant personality traits in relation to change over time in health, well-being, stress and indicators of the psychosocial work environment

Multilevel growth curves were performed to investigate if a) there were changes in the outcome variables over time (level 1), and b) if possible changes could be explained by health-relevant personality traits (level 2) examining cross-level interactions. The results revealed that seven of the 11 outcome variables significantly changed over time. For three of these outcomes, the changes were predicted by health relevant personality-traits. For four outcome variables, there was significant variance over time, meaning that individuals developed differently. A detailed description of these results is presented in the following section.

Concentration ability, energy, and sense of control improved over time. These improvements were partly explained by higher baseline levels of negative affectivity (Concentration ability: *B* 0.055, p < 0.01; Energy: *B* 0.038, p < 0.05; Sense of control: *B* 0.072, p <0.01). This means that individuals who exhibited higher levels of negative affectivity at baseline improved more over time in relation to these outcomes. For self-rated health, job satisfaction, work atmosphere and social support, there was significant slope variance, meaning that individuals developed differently over time. For job satisfaction, the variance over time was partly explained by negative affectivity (*B* 0.045; p < 0.05) and alexithymia (*B* 0.061; p < 0.01). The variance over time in work atmosphere (*B* 0.046; p < 0.05) and social support (*B* 0.049; p < 0.05) were partly explained by negative affectivity. For self-rated health, none of the personality traits explained the variance over time, implying that the variance over time was explained by something not accounted for in these analyses.

Some of the changes in outcomes were not predicted by health-relevant personality traits. Sleep quality (Mean 0.029; p < 0.001) and work efficiency (Mean 0.063; p < 0.001) improved, and workload (Mean 0.070; p < 0.001) and stress (Mean 0.125; p < 0.001) increased over time, but these changes were not predicted by the health-relevant personality traits.

### Prediction of typical ratings

As part of the multilevel growth curve analyses, individuals’ typical ratings of health, well-being, stress and indicators of the psychosocial work environment were assessed. Typical ratings refer to how an individual commonly tends to respond to a certain question when assessed repeatedly. The section below describes health-relevant personality traits as predictors of how individuals typically scored on the HW-11 items.

Typical ratings of health, well-being and the psychosocial work environment were positively predicted by hedonic capacity. This means that those with higher levels of hedonic capacity typically rated better health (*B* 5.823; p < 0.001), sleep (*B* 7.312; p < 0.001), concentration ability (*B* 6.022; p <0.001), more energy (*B* 5.254; p < 0.001) and sense of control (*B* 8.063; p <0.001) compared to those with lower levels of that trait. Furthermore, these individuals tended to be more satisfied with their social support (*B* 13.927; p < 0.001), perceive more job satisfaction (*B* 8.546; p < 0.001), higher work efficiency (*B* 6.933; p < 0.001) and higher workload (*B* 4.144; p < 0.05) as well as better work atmosphere (*B* 5.544; p < 0.01). The only variable that was not significantly related to hedonic capacity was stress. This means that how an individual typically responded to the item about perceived stress was not predicted by hedonic capacity.

The typical ratings of the HW-11 items were negatively predicted by negative affectivity. Thus, individuals with higher levels of negative affectivity typically rated poorer health, well-being and more stress compared to those with lower levels of that trait. (Concentration ability: *B* -6.179, p < 0.001; Sense of control: *B* -5.727, p < 0.001; Energy: *B* -5.969, p < 0.001; Sleep: *B* -6.241, p < 0.001; Self-rated health: *B* -4.971, p < 0.001; Social support: *B* -4.553, p < 0.001; Stress: *B* 6.734, p < 0.001). They also typically rated the psychosocial work environment as worse (Job satisfaction: *B* -5.352, p < 0.001; Work atmosphere: *B* -4.095, p < 0.01; Work efficiency: *B* -3.874, p < 0.01) compared to those with lower levels of that trait. Antagonism negatively predicted individuals’ typical ratings of energy (*B* -2.780; p < 0.05), sense of control (*B* -2.955; p < 0.05) and job satisfaction (*B* -3.538; p < 0.05). This means that highly antagonistic persons typically rated lower energy levels, less control and lower job satisfaction compared to those who were less antagonistic. Impulsivity negatively predicted sleep quality (*B* -2.555; p < 0.05) and concentration ability (*B* -2.030; p < 0.05). Thus, highly impulsive individuals typically rated poorer sleep and worse ability to concentrate compared to those with lower levels of that trait.

## Discussion

The aim of this study was to investigate health-relevant personality in relation to repeated assessments of health, well-being, stress and indicators of the psychosocial work environment. Figs 2–6 clearly demonstrate fluctuations in the outcome variables over time, including pronounced seasonal variations in responses. There also seem to be systematic and continuous differences in absolute rating levels between those with low, medium and high levels of the health-relevant personality traits. Furthermore, there were significant changes over time for seven outcome variables and significant slope variance (i.e., variation) over time for the remaining four outcomes. Improved concentration ability, energy level and sense of control were partly explained by personality traits. Increases in sleep quality, work efficiency, workload and stress were attributed to something other than the health-relevant personality traits. There was significant variance over time in perceived job satisfaction, work atmosphere and social support, which was partly explained by negative affectivity. There was also significant variance over time in self-rated health, but this was not explained by health-relevant personality traits. Individuals’ typical ratings of HW-11 were predicted by hedonic capacity, negative affectivity, impulsivity and antagonism. To our knowledge, this is the first study to investigate health-relevant personality traits in relation to repeated assessments of health, well-being, stress and psychosocial work environment indicators.

### Seasonal variations in trends over time

There were apparent seasonal variations in all outcome measures. Most health-related indicators improved during the summer months, with peaks in August when the participants returned to work after their vacation. Perceptions of the psychosocial work environment also peaked in August and declined during the fall. Similar findings have been reported for self-rated health, energy and stress [7] as well as for happiness [8]. Thus, seasonal variation is clearly a confounder that needs to be considered when assessing indicators of health, well-being and the psychosocial work environment.

Seasonal variations as indicators of the psychosocial work environment have, to our knowledge, not been investigated previously. These novel findings are therefore important. They have implications for the utilization, reliability and accurate interpretation of results from questionnaires used in organizational development and occupational health promotion. Considering that action plans and interventions can be based on the survey outcomes, it is critical to better understand the dynamics and volatility of such measures. If an annual occupational survey is conducted in November and actions implemented in May or August, the situation may have changed notably. There is a risk that improvement efforts will be outdated, fail or be questioned by employees and managers who do not recognize the situation. Furthermore, indicators of improvement or worsening may just be the result of a natural variation over time, which can make interventions unnecessary. Seasonal variations can obviously be a confounder in survey responses. This aspect needs to be further problematized, studied and elaborated on in future organizational development and occupational health promotion research. Our results, combined with previous research, emphasize the importance of regular assessments that can properly account for temporary fluctuations in outcome variables and the possibly confounding effect of seasonality. Organizational assessments and thereto following interventions and action plans should consider these naturally occurring variations over time. Trends, rather than the results of single assessments, may provide a more valid foundation for possible action plans and interventions.

### Health-relevant personality in relation to survey responses

In addition to the seasonal variations in responses, this study found that health-relevant personality predicted individuals’ typical ratings of health, well-being, stress and the psychosocial work environment. The most consistent findings were noted for hedonic capacity (a facet of extraversion) and for negative affectivity (a facet of neuroticism). Individuals with higher levels of hedonic capacity typically rated better health, well-being and psychosocial work environment compared to those with lower levels of that trait. These findings are in a sense logical and in line with previous research. For instance, extraversion has been associated with better health [19] and more job satisfaction [36, 40]. The present findings align with other studies and the longitudinal design with repeated measures over a consecutive time period adds to the robustness of the previously demonstrated associations between positive dimensions of personality, health and aspects in the psychosocial work environment.

Our findings also demonstrate that those with higher levels of negative affectivity typically rated poorer health, well-being, more stress and psychosocial work environment compared to those with lower levels of that trait. This finding is also in line with several prior studies [13–15, 18–21, 24–26, 28–30, 33, 35–40]. Several possible explanations for these associations have been proposed previously but general conclusions are difficult to draw since there are contextual, social, dispositional and other factors to consider.

Interestingly, the present study found that improvements in concentration ability, energy and sense of control could partly be explained by higher initial levels of negative affectivity. There may be several explanations for this. One possibly contributing factor refers to the intervention itself, which included individual feedback on the responses and self-help components. A study of intervention adherence, that we conducted on the same sample, presented a finding that may corroborate this assumption. We found that negative affectivity positively predicted the utilization of self-help components, at least for women [46]. Higher utilization could in turn be attributed to poorer perceived health among those who scored higher levels of negative affectivity. Thus, individuals with poorer baseline ratings may have been more motivated to intervene during the course of the study, in order to improve the situation. This could perhaps partly explain the associations between improvements over time for those with higher initial levels of negative affectivity.

Hedonic capacity predicted typical workload ratings positively, but not typical ratings of stress. Conversely, higher levels of negative affectivity predicted higher typical ratings of stress levels, but not typical ratings of workload. The reasons for these results may be confusing at first, since high workload is commonly considered to be related to high stress levels. However, both stress and workload are variables that could be interpreted in a multitude of ways. Hence, high workload and stress could be considered to be something positive by some and negative by others [47]. Obviously, in this sample and for the highly hedonic persons, higher typical workload ratings are combined with other positive ratings on indicators of health and wellbeing. Higher typical workload ratings could therefore be an indicator of better perceived health among these individuals. This is also confirmed in a study assessing workload in relation to health assessments over time [47]. So, higher typical workload ratings can be explained by better health, meaning that good health could, at least for some, be a prerequisite for enduring high workload over time.

Parts of these results can possibly be explained by three well-established psychosocial work environment models, i.e., a) Demand-Control model, b) Effort-Reward Imbalance model and c) Job Demand-Resources model. The basic idea in the Demand-Control model suggests that high demands in combination with low control increases the risk for job strain and ill-health [48, 49]. A combination of high demands and high control characterizes an active and healthy work situation. This can be related to the present findings where those with higher hedonic capacity rated better health, perceived more control, were more satisfied with their jobs and rated higher workload (demands). Similarly, the Effort-Reward Imbalance model [50] stipulates that individuals can handle and benefit from investing high efforts if they perceive that they obtain matching rewards. It is plausible that individuals who are highly hedonic to a larger extent perceive that their efforts pay off and are rewarded properly, for instance by achieving goals, etc. Conversely, it is also plausible that appropriate rewards may yield higher levels of hedonic capacity. This could also partly explain why highly hedonic individuals perceived higher workload, which, for them, might be experienced as something positive. This conclusion is also corroborated by a recent study suggesting that workload is a multidimensional construct, indicating that the appraisal (satisfaction or dissatisfaction) of workload is highly associated with health- and work environment-related outcomes [47]. Lastly, the Job Demands-Resources model suggests that high job demands in combination with sufficient resources (either personal or organizational) entail better health and lower risk for stress-related problems, such as burnout [51–53]. In accordance with the other models, this one also suggests that high job demands in combination with high resources is associated with more job satisfaction as well as higher efficiency and work engagement. Relating our findings to this model, it is plausible that individuals with higher levels of hedonic capacity perceive that they have more or better personal and organizational resources. This could in turn act as a buffer for potentially negative effects of high workload over time, i.e., high job demands. These well-established models can probably explain the results to some extent. However, considering the multitude of possible causal, mediating and interacting variables, this topic needs to be further elaborated on in future studies.

### Study strengths and limitations

This study has both strengths and limitations. A major strength was the longitudinal study design with repeated measurements over 20 consecutive months. To our knowledge, such large-scale studies with repeated measures of health, well-being, stress and aspects in the psychosocial work environment have not been conducted before with employees in similar work settings (schools). The repeated assessments and the phrasing of them systematically aimed to capture the current state instead of retrospective recollections. Since recall bias commonly constitutes a limitation in studies, repeated measures of the current state most likely minimize the risk of drawing incorrect conclusions about natural variability in these outcomes over time. This strategy also yielded the finding of systematic fluctuation and seasonality in outcome variables. It would have been interesting to assess if, and to what extent the outcomes with the repeated assessments in this study differ from similar studies using retrospective ratings with possible recall bias. To our knowledge, there are no comparable studies, which makes it difficult to assess if and how much recall bias may generally influence results. There are however studies suggesting that recall bias may influence responses to health-related questions [54]. For example, a literature review concluded that associations were stronger when using momentary assessments (i.e., similar to the repeated measures used in the present study), compared to retrospective measurements. Thus, for items such as self-rated health, stress, etc., which involve biological responses, repeated measures seem to capture a more accurate evaluation compared to retrospective ratings that are prone to recall bias and false memory. However, assessments of general beliefs about oneself, such as personality traits, seem to be less affected by recall bias since the respondent’s perception is not limited to a specific time and place [54].

Another strength of this study was the design of the response scales that were used. Verbal rating scales (VRS) and visual analogue scales (VAS) were used in favor of for instance Likert scales. VAS scales have been found to have several advantages compared to Likert scales in some circumstances, particularly when it comes to assessing variables that typically fluctuate over time [55]. For instance, in most scales, there is a risk for end-aversion bias. This means that respondents tend to avoid the end response alternatives on a rating scale. This may sometimes be a limitation, especially when using 5-graded Likert scales aiming at measuring changes over time for instance. End-aversion bias may leave only the three response alternatives as a result, which limits the ability of this scale type to assess real fluctuation of a variable momentarily and over time. The VRS, ranges from 0–100 and includes five descriptors that were evenly distributed along the scale. Consequently, it combines the strengths of the VAS and Likert, by allowing for both variance and understanding what each rating actually means. End aversion bias will probably still exist but be negligible considering the continuous properties of the VAS. The fluctuations of variables momentarily and over time are more precisely and accurately captured when using VRS that enable a more proper assessment of variations over time.

The selection of personality inventory is not always an easy choice, and the selection of HP5i in assessing health-relevant personality traits can be discussed. This inventory is considerably less frequently used than for instance NEO-PI [56], which limited the possibility of relating the present findings to previous research. However, there is no gold standard as to which personality inventory to use. There have been scientific discussions regarding the application of broader traits to capture wide personality dimensions, such as extraversion, neuroticism, etc., or specific traits or facets [28, 42]. There are advantages and disadvantages with both approaches and in the present study, using specific traits was feasible for several reasons. Firstly, the study constituted part of a large-scale health promotion and stress management intervention and thus, it made sense to assess personality traits that are related to health and well-being. Secondly, a short scale was feasible for practicality. The study participants’ work situation and conditions limited their ability to respond to lengthy questionnaires. In order to encourage participants to respond, a brief 20 item personality inventory was more feasible instead of a lengthy one. The psychometric properties in HP5i were modest but acceptable, as has also been found elsewhere [22, 57]. There were also weak to moderate correlations between the HP5i items (see Table 1), and most of them in line with what has been reported elsewhere [22, 57]. However, there may have been patterns within the personality traits that we did not consider in the present study. For instance, analyzing how the health-relevant personality traits interact in an individual could perhaps yield even more precise prediction models. It is plausible that, for instance, high negative affectivity combined with high hedonic capacity and alexithymia may prove to buffer or worsen outcomes. Future studies should assess the potential differences in outcomes when comparing the numerous possible combinations using patterns of traits with the results of isolating each trait.

The present study did not account for other possible confounders that may have influenced the results. In future publications, analyses will be conducted that assess possible confounders such as for example gender, age, socioeconomic status, coping strategies and other environmental-, health- and work-related variables. Such analyses were not within the scope of this study as the focus was on personality. However, including such variables in future studies would be valuable for both theory and practice. It would also be interesting to assess seasonal variations in other organizations than school staff. Unpublished data from the same country shows similar patterns with regard to the seasonal variation, and future studies should assess if and how seasonal variation exists in other types of organizations, countries and regions.

A possible limitation of this study was that the low and time-variant response rate might imply selection bias. It is possible that individuals who were generally more interested in the intervention study responded to HW-11 and are thus over-represented the study population. However, we consider this risk to be relatively low given the large variability in the outcomes for each assessment point. The perceptions of health, well-being, stress and aspects of the psychosocial work environment ranged from the lowest response alternative (0) to the highest (100); e.g. very poor self-rated health to very good self-rated health (except for some outcomes in July). Furthermore, the distribution of the responses in for instance self-rated health were comparable to a national population sample [58], which indicates that the study participants constituted a representative sample. The number of assessments decreased the month of July in both years of the study, and this was due to the yearly vacation period that most of the participants had. However, some participants worked during those months in daycare. Unfortunately, we do not know how many of the responses in July that represent individuals who worked and how many that represent individuals who were on vacation. Responding to the assessments during the vacation was optional. Considering that the participants received individual feedback on their responses, this might have encouraged some participants to respond to all or some of the HW-11 items while on vacation too. For instance, since participants were able to view their development over time, they could compare their ratings during the summer with the rest of the year.

In summary, the risk for selection bias due to attrition is considered to be relatively low for the reasons stated above, and due to the application of the method Full Information Maximum Likelihood (FIML) to adjust for potential systematic attrition. However, other methods, such as Inverse Probability Weighting (IPW) or Non-Response Cell Weighting (NRCW) [59], could have been applied. Given that there are both advantages and disadvantages with these methods, the present study applied FIML in favor of weighting-based approaches, due to the nature of the data and the study purpose.

### Practical implications

The findings in the present study have several practical implications. Firstly, a better understanding and awareness of natural fluctuation and seasonal variations in the outcome variables may facilitate the interpretation of survey responses. This could be a way to decrease the risk of drawing incorrect conclusions about what the outcomes represent. Furthermore, naturally occurring seasonal variations could also be important to consider when implementing action plans based on survey responses. If action plans, to for instance improve job satisfaction, are implemented several months after the survey was distributed, the situation might have changed and needs to be re-assessed. For example, if job satisfaction was found to be low in November, actions might be taken during March or April. A new assessment made in May might indicate higher levels of job satisfaction. However, given the seasonal variation in responses, the higher levels of job satisfaction could be natural and expected in May, and it is difficult to know if they are related to actions that were implemented. Consequently, to facilitate the interpretation of variations in health and psychosocial work environment aspects, regular assessments seem to have several benefits in practical terms. Secondly, taking health-relevant personality into consideration when interpreting the results and implementing action plans can be of practical importance for leaders, human resources, organizational developers, and researchers. Considering that our perceptions clearly vary naturally or seasonally, and also differ due to individual characteristics, the need to better understand what the ratings actually mean is prominent. Just combining all the results to mean values that do not consider health-relevant personality traits may bias the results favorably for some and detrimentally for others. Some issues will likely be overestimated and some underestimated. Understanding the differences, for instance that, for some individuals, high perceived job satisfaction may entail for instance high workload will reduce the risk of flawed interpretations and pointless interventions. With a better understanding of and consideration to these aspects, more relevant, possibly tailored interventions based on needs and correct interpretations of outcomes could be implemented. Such interventions would hopefully yield more favorable outcomes.

## Conclusions

Health-relevant personality traits seem to be associated with changes in indicators of health, well-being, stress and the psychosocial work environment over time. The findings of the present study highlight the importance of regular assessments of indicators of health, well-being, stress and the psychosocial work environment. These measures clearly vary over time and the trends over time seem to follow a pronounced seasonal pattern. A better understanding of the trends over time may be highly relevant for organizations, where actions or interventions are often implemented based on retrospective ratings. Seasonal variations are also relevant to consider as a possible confounder in longitudinal research studies using repeated assessments.
